# The geometric model of the human mitral valve

**DOI:** 10.1371/journal.pone.0183362

**Published:** 2017-08-25

**Authors:** Xiaoqin Shen, Tiantian Wang, Xiaoshan Cao, Li Cai

**Affiliations:** 1 School of Sciences, Xi’an University of Technology, Xi’an, 710054, P.R.China; 2 State Key Laboratory of Transducer Technology, Chinese Academy of Sciences, Shanghai, 200050, P.R.China; 3 NPU-UoG International Cooperative Lab for Computation & Application in Cardiology, Northwestern Polytechnical University, Xi’an, 710072, P.R.China; Nanjing University of Aeronautics and Astronautics, CHINA

## Abstract

The mitral valve, which lies between the left atrium and the left ventricle, plays an important role in controlling the uniflux of blood from the left atrium to the left ventricle as one of the four human heart valves. A precise description of the shape of human mitral valve has vital significance in studying its physiological structure and periodic movement. Unsatisfyingly, there is almost no unified mathematical description of the same shape of human mitral valve in literature. In this paper, we present a geometric model for human mitral valve, as an elastic shell with a special shape. Parametric equations for the shape of human mitral valve are provided, including the anterior and the posterior parts, which can be thought as portions of two interfacing semi-elliptic cylindrical shells. The minor axis of one ellipse is equal to the major axis of the other. All the parameters are determined from the statistical data. Comparison of fitting results with existing examples validates the accuracy of our geometric model. Based on the fitting shape, one can further simulate the physiological function of the mitral valve using a suitable dynamic physical equation.

## Introduction

Research shows that the heart valves open and close about 3 × 10^9^ times in a human lifetime [1]. Because of this highly frequent movement, the human heart valves are subject to all kinds of diseases, for example, valve regurgitation congruent disease which can lead to cardiac dysfunction. Therefore, the study of physiological structure and periodic movement is vitally significant.

The mitral valve, located between the left atrium and the left ventricle [2], is one of four important human heart valves which periodically open and close under the differential pressure of the heart and blood coupling [3]. It plays an important role in maintaining the uniflux of blood from the left atrium to the left ventricle. Therefore, the study of the mitral valve’s shape has become one of the key research topics in cardiovascular system simulation. This study will provide important theoretic and application support for the analysis of the pathological structure of mitral valve insufficiency and prosthesis.

How to accurately describe the shape of human mitral valve leaflets mathematically? There are some literatures relating to this field. Kunzelman *et al*. [4] first mimicked valve behavior during closure on the basis of detailed data but oversimplified the structure of the mitral valve. Then, Kunzelman *et al*. [5] compared the size of the human mitral valve with a porcine valve. However, they did not divide the posterior leaflets into three subregions. Schneider *et al*. [6, 7] developed an automatic pipeline for mitral valve geometry delineation from transesophageal echocardiography (TEE) images. By using machine learning to detect the mitral valve on 3D TEE or CT data, Gao *et al*. [8] summarized the state-of-the-art modelling of the mitral valve, including static and dynamics models with fuid-structure interaction. Ma *et al*. [9] deduced an anatomical model of a human mitral valve based on in vivo magnetic resonance imaging. Ranganathan *et al*. [10] reported the size and morphology of the human mitral. Liu *et al*. [11, 12] proposed a novel view centralized multi-atlas classification method, which also provided us a useful method to determine the shape of the human mitral valve. However, these authors did not provide parametric equations for its shape, and there is almost no unified mathematical description of the same shape of human mitral valve. Therefore, it is important to propose the fitting function to describe the human mitral valve’s shape for both function simulation of the human mitral valve and for prosthetic mitral valve repair.

In this paper, we describe the shape of the human mitral valve leaflets and provide parametric equations based on statistical data of the human mitral valve. The main research contents are as follows: (i) based on the structure and size of the human mitral valve, we study the mitral valve as an elastic shell; (ii) based on the morphological characteristics of the mitral valve during its entire opening, we divide the mitral valve into the anterior and the posterior parts, each of which are treated as a portion of the semi-elliptic cylindrical shell and are provided with parametric equations, respectively; (iii) based on the available and specific statistical data of the human mitral valve, we determine all the parameters in fitting functions; (iv) we visualize the parametric equations of the human mitral valve; and (v) we verify the accuracy of the fitting function by comparing the computation of our parametric equations with the statistical data.

## Fitting functions of the human mitral valve

### The structure of the human mitral valve

In order to fit the parametric equations of the human mitral valve’s shape, we should clearly know the basic morphology and structure of the human mitral valve.

The human mitral apparatus is composed of four principal parts: valve leaflets, mitral annulus, papillary muscles and chordae tendineae. The mitral annulus is divided into two sections named the anterior annulus and the posterior annulus according to its special morphology. Mitral leaflets are also divided into two sections named the anterior leaflet and the posterior leaflet, which are attached to the anterior annulus and the posterior annulus, respectively [13]. The occlusion and separation of these two parts determine the mitral valve’s opening and closing. Mitral valve apparatus has two independent papillary muscles, which can be viewed as two independent nodes. There are 60 chordae tendineae linking the papillary muscles, valve leaflets and annulus. Papillary muscles and chordae tendineae are closely related to mitral valve stress, whereas the mitral leaflets and annulus determine the shape of the mitral valve.

### The shape of the human mitral valve-leaflets

**Fig 1:**
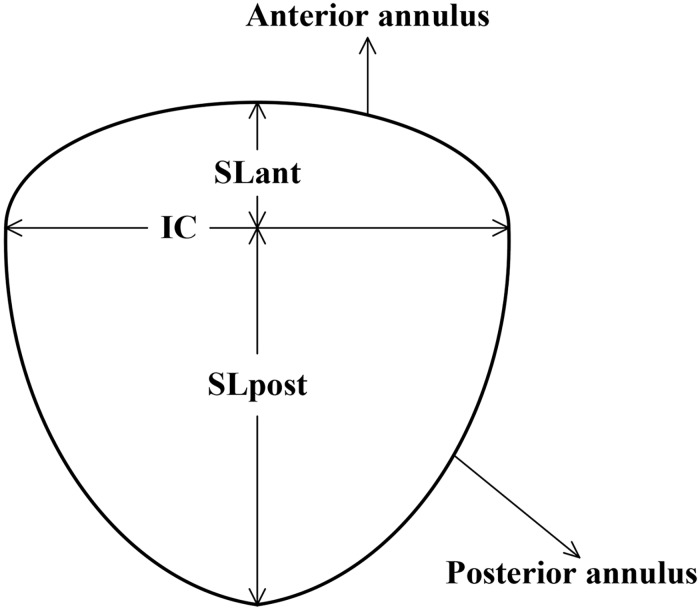
The mitral annulus. The inter-commissural axis IC = 2.8 cm, The septolateral of the anterior SL_*ant*_ is equal to 1.04 cm and the septolateral of the posterior SL_*post*_ is equal to 2.11 cm.

Fig 1 shows an anatomical drawing of the two parts of the human mitral valve, i.e., the anterior leaflet and the posterior leaflet. It is clear that the anterior leaflet comprises a single wide cusp whereas the posterior leaflet comprises three narrow cusps, namely the subregions P1, P2 and P3 [14, 15]. Based on the geometric characteristics of the valve leaflets, we will study the anterior leaflet and the posterior leaflet, respectively.

In [16], the anterior leaflet and posterior leaflet attached to the anterior annulus and posterior annulus, respectively, can be viewed as a portion of two interfacing semi-elliptic cylinders. The anterior and posterior tracts could be described as two semi ellipses sharing the inter-commissural (IC) axis (cf. Fig 1). By properly scaling values we give IC = 2.8 cm. The septolateral of the anterior (SL_*ant*)_ is equal to 1.04 cm and the septolateral of the posterior (SL_*post*)_ is equal to 2.11 cm, respectively. Thus, the septolateral diameter is obtained by 3.15 cm, which is consistent with the data provided by Timek *et al*. [17].

Based on the above analyses, we can summarize as follows: (i) the human mitral valve can be viewed as a portion of two interfacing semi-elliptic cylindrical shell. The minor axis of the ellipse is equal to the major axis of the other two semi-ellipses sharing the IC axis in the xy plane; (ii) the anterior leaflet can be thought as a wide cusp, and the boundary shape can be fitted by sine function; and (iii) the posterior leaflet can be deemed as three narrow cusps, and the boundary shape looks like three end-to-end sine curves.

### Parametric equations for mitral valve leaflets

**Fig 2:**
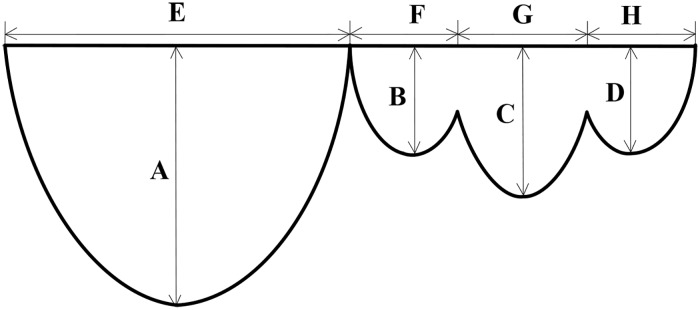
Diagram of the mitral valve. The height of the anterior leaflet and the posterior leaflet (P1, P2, P3) is A, B, C and D, and the width of the them is E, F, G and H.

In this section we will provide parametric equations for the anterior leaflet and posterior leaflet, respectively. Accurate and detailed statistical data of the human mitral leaflets obtained by the authors was shown in Table 1 [10], which records data 50 healthy humans’(including 26 men and 24 women, aged vary from 15 to 85 years old). In order to determine all the parameters of the parametric equations for the mitral valve, we prepared a diagram (cf. Fig 2) on the basis of Table 1, where A, B, C, D, E, F, G and H take values in Table 2.

**Table 1:** Statistical data of mitral leaflets.

Width and Height	Width(cm)	Height(cm)
Anterior leaflet	3.6(2.5-4.8)	2.4(2.0-3.0)
P1	1.6(0.9-4.0)	1.1(0.9-2.0)
P2	2.3(1.3-3.8)	1.4(0.9-2.0)
P3	1.5(0.9-3.1)	1.0(0.6-1.7)

**Table 2:** Width and height of mitral leaflets.

Width	E = 3.6 cm	F = 1.6 cm	G = 2.3 cm	H = 1.5 cm
Height	A = 2.4 cm	B = 1.1 cm	C = 1.4 cm	D = 1.0 cm

#### Parametric equation for the anterior leaflet

As we all know, the parametric equation of the whole elliptic cylinder is given as follows:

$$\begin{array}{c} \vec{\theta }\left(y_{1},y_{2}\right)=\left(a\text{ cos }y_{1},b\text{sin}y_{1},hy_{2}\right), \end{array}$$ (2.1)

where *y*_1_ ∈ [0, 2*π*], and *y*_2_ ∈ [0, 1] are variables, *a* and *b* are coefficients representing the semi-axes of the ellipse and *h* is the maximum height of the elliptic cylindrical shell.

**Fig 3:**
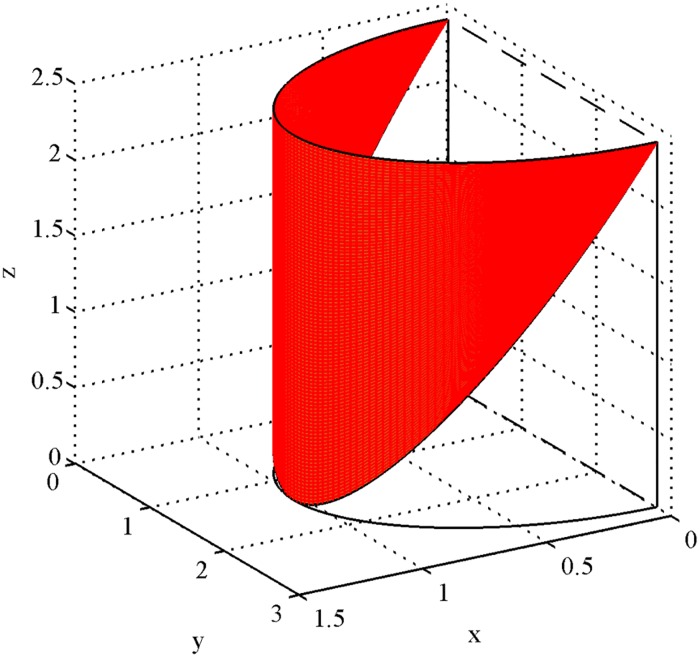
The anterior leaflet. The anterior leaflets is deemed as a portion of the semi-elliptic cylinder shell which is cut along the major axis of the xy plane.

The anterior leaflets is deemed as a portion of the semi-elliptic cylinder shell [18] which is cut along the major axis of the xy plane (cf. Fig 3). We have to determine the value of every coefficient including *a*, *b* and *h*, and the rang of every variable including *y*_1_ and *y*_2_, to fit the shape of the anterior leaflet.

How to determine the coefficients a, b, and h? According to the statistical data of the anterior annulus [16], as shown in Fig 1, we take IC = 2.8 cm which represents the major axis of the anterior annulus. Thus, $a=\frac{1}{2}\text{IC}=1.4\text{ cm}$. And SL_*ant*_ = 1.04 cm is the minor semi-axis of the ellipse which represents the posterior annulus. Thus, we derive *b* = 1.04 cm. Similarly, we can take *h* = 2.4 cm as the maximum height of the anterior leaflet from Table 1.

**Fig 4:**
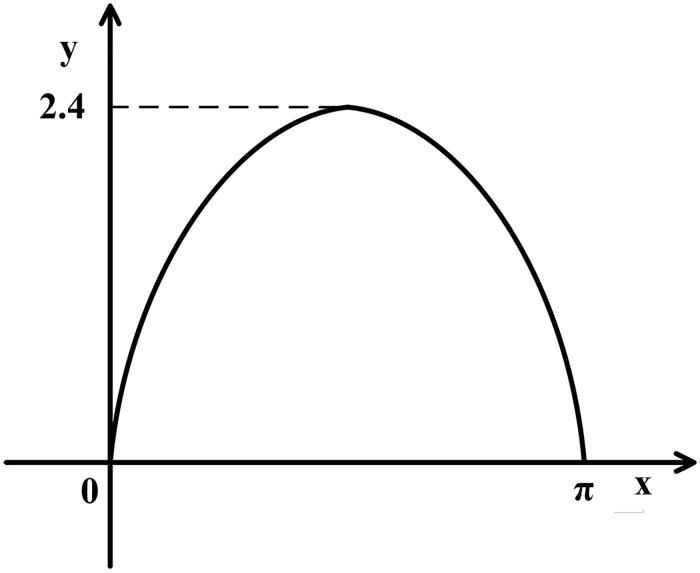
The boundary curve of the anterior leaflet. The value range of the variable *y*_1_ is [0, *π*], and the range of the variable *y*_2_ is [0, sin *y*_1_].

How to determine the range of variables *y*_1_ and *y*_2_? We have prepared a diagram of the anterior leaflet boundary in a plane rectangular coordinate system (cf. Fig 4). We choosing the sine of half a period to simulate the shape of the anterior leaflet on the basis of the shape. The value range of the variable *y*_1_ is [0, *π*] because the anterior leaflet is a semi-elliptic cylinder shell. As shown in Fig 4., owing to the shape of the anterior leaflet, the range of the variable *y*_2_ is [0, sin *y*_1_].

Therefore, we obtain the fitting function for the anterior leaflet defined as follows:

$$\begin{array}{c} \vec{\theta }\left(y_{1},y_{2}\right)=\left(1.4\text{ cos }y_{1},1.04\text{ sin }y_{1},2.4y_{2}\right), \end{array}$$ (2.2)

where *y*_1_ ∈ [0, *π*], and *y*_2_ ∈ [0, sin *y*_1_].

#### Parametric equation for the posterior leaflet

**Fig 5:**
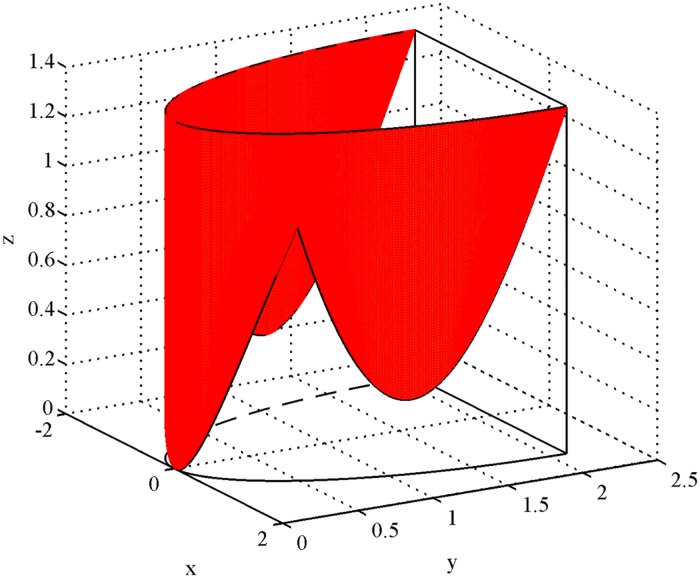
The posterior leaflet. The posterior leaflet is deemed a portion of the semi-elliptic cylinder shell which is cut along the minor axis of the xy plane.

The posterior leaflet is deemed a portion of the semi-elliptic cylinder shell which is cut along the minor axis of the xy plane (cf. Fig 5). We divided the boundary of the posterior leaflets into three sections (three subregions P1, P2, P3), which are simulated by three sine functions, respectively.

How to determine the coefficients a, b, and h? According to the statistical data of the posterior annulus [16], as shown in Fig 1, we take IC = 2.8 cm which represents the minor axis of the posterior annulus. Thus, $\text{a}=\frac{1}{2}\text{IC}=1.4\text{ cm}$. And SL_*post*_ = 2.11 cm is the major semi-axis of the ellipse which represents the posterior annulus. Thus, we derive *b* = 2.11 cm. Similarly, we can take h equaling 1.1 cm, 1.4 cm, 1.1 cm as the height of three subregions from Table 1, respectively. We can calculate the interval of the variate *y*_1_ for the three subregions according to the proportion of their respective width to the total annulus. The width of the three subregions is F = 1.6 cm, G = 2.3 cm, H = 1.5 cm, summation is F+G+H = 5.4 cm, hence the interval of *y*_1_ is $y_{1}\in \left[\pi ,\frac{35}{27}\pi \right)$ for P1, $y_{1}\in \left[\frac{35}{27}\pi ,\frac{93}{54}\pi \right)$ for P2, $y_{1}\in \left[\frac{93}{54}\pi ,2\pi \right)$ for P3, respectively. Summation of three intervals should just equal to[*π*, 2*π*].

**Fig 6:**
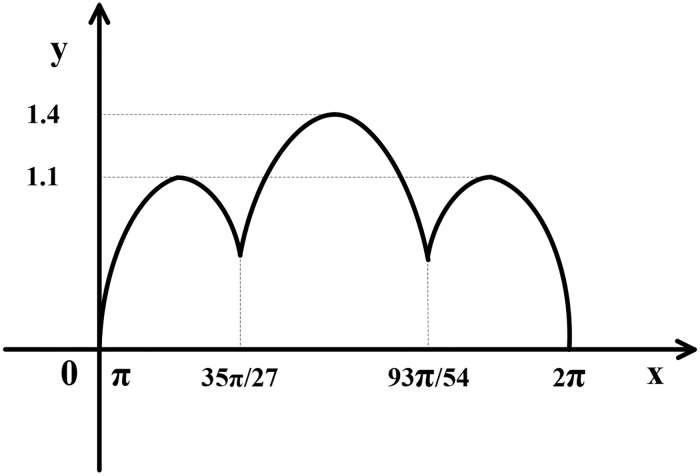
The boundary curve of the posterior leaflet. The value ranges of variables of P1 are $y_{1}\in \left[\pi ,\frac{35}{27}\pi \right)$ and *y*_2_ ∈ [0, sin 3(*y*_1_ − *π*)). The value ranges of variables of P2 are $y_{1}\in \left[\frac{35}{27}\pi ,\frac{93}{54}\pi \right)$ and $y_{2}\in \left[\frac{11}{14}\text{sin}\frac{\pi }{9},\frac{11}{14}\text{sin}\frac{\pi }{9}+\left(1-\frac{11}{14}\text{sin}\frac{\pi }{9}\right)\text{sin}\left[\frac{54}{23}\left(y_{1}-\frac{35}{27}\pi \right)\right]\right))$. The value ranges of variables of P3 are $y_{1}\in \left[\frac{93}{54}\pi ,2\pi \right]$ and $y_{2}\in \left[0,\text{sin}\frac{16}{5}\left(2\pi -y_{1}\right)\right)$.

In order to determine the range of the *y*_2_ we have prepared a graph of the posterior leaflet boundary along the z-axis in a plane rectangular coordinate system (cf. Fig 6) which are simulated by three sine functions, respectively. The value range of the variable *y*_2_ is *y*_2_ ∈ [0, sin 3(*y*_1_ − *π*)] for P1, $y_{2}\in \left[\frac{11}{14}\text{sin}\frac{\pi }{9},\frac{11}{14}\text{sin}\frac{\pi }{9}+\left(1-\frac{11}{14}\text{sin}\frac{\pi }{9}\right)\text{sin}\left[\frac{54}{23}\left(y_{1}-\frac{35}{27}\pi \right)\right]\right]$ for P2, $y_{2}\in \left[0,\text{sin}\frac{16}{5}\left(2\pi -y_{1}\right)\right]$ for P3, respectively.

Thus, we have obtained the parameter equation of P1 as follows:

$$\begin{array}{c} \vec{\theta }\left(y_{1},y_{2}\right)=\left(1.4\text{ cos }y_{1},2.11\text{ sin }y_{1},1.1y_{2}\right), \end{array}$$ (2.3)

where $y_{1}\in \left[\pi ,\frac{35}{27}\pi \right)$, and *y*_2_ ∈ [0, sin 3(*y*_1_ − *π*)).

Next, we have obtained the parameter equation of P2 as follows:

$$\begin{array}{c} \vec{\theta }\left(y_{1},y_{2}\right)=\left(1.4\text{ cos }y_{1},2.11\text{ sin }y_{1},1.4y_{2}\right), \end{array}$$ (2.4)

where $y_{1}\in \left[\frac{35}{27}\pi ,\frac{93}{54}\pi \right),$ and $y_{2}\in \left[\frac{11}{14}\text{sin}\frac{\pi }{9},\frac{11}{14}\text{sin}\frac{\pi }{9}+\left(1-\frac{11}{14}\text{sin}\frac{\pi }{9}\right)\text{sin}\left[\frac{54}{23}\left(y_{1}-\frac{35}{27}\pi \right)\right]\right))$.

Finally, we have obtained the parameter equation of P3 as follows:

$$\begin{array}{c} \vec{\theta }\left(y_{1},y_{2}\right)=\left(1.4\text{ cos }y_{1},2.11\text{ sin }y_{1},1.1y_{2}\right), \end{array}$$ (2.5)

where $y_{1}\in \left[\frac{93}{54}\pi ,2\pi \right],$ and $y_{2}\in \left[0,\text{sin}\frac{16}{5}\left(2\pi -y_{1}\right)\right)$.

#### Coupling

**Fig 7:**
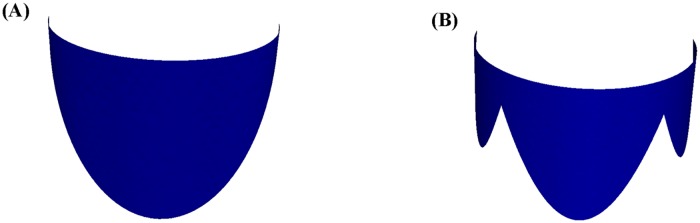
Visualization results. (A) is the visualization result of the anterior leaflet, and (B) is the visualization result of the posterior leaflet.

**Fig 8:**
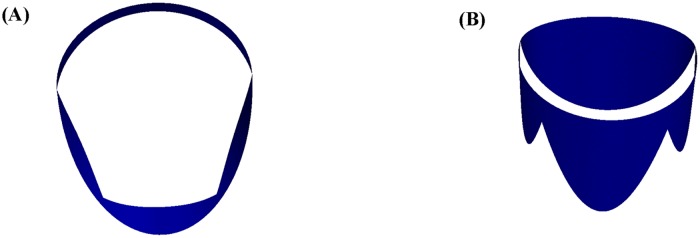
Coupling results. (A) is the vertical view of the coupling results, and (B) is the front view of the coupling results.

We have visualized the results of the function fitting for the human mitral valve leaflets, i.e., the anterior leaflet (cf. Fig 7(A)) and the posterior leaflet (cf. Fig 7(B)). Ideally, the two graphs can be coupled since the major axis of the anterior annulus is just equal to the minor axis of the posterior annulus. Ultimately, we obtained a total graph of the human mitral valve from different angles of view, i.e., vertical view (cf. Fig 8(A)) and side view (cf. Fig 8(B)). In addition, we verified the correctness of the parametric equations. We compute the height of the anterior leaflets and posterior leaflets by our parametric equations. Then we compare these data with [5], [19] and [20] in Table 3. It is clear that all of these data are within the range, which verifies the correctness of the parametric equations for the human mitral valve.

**Table 3:** Comparison of our methods with those of Kunzelman[5], Cheichi[19] and Rusted[20] on the height of mitral leaflets.

Workers	Anterior leaflet(cm)	P1(cm)	P2(cm)	P3(cm)
Our results	2.4	1.1	1.4	1.1
Kunzelman	2.0(1.6-2.4)	Not mentioned	1.2(1.0-1.4)	Not mentioned
Cheichi	2.1(1.9-3.2)	1.1(0.8-1.8)	1.4(1.0-2.5)	0.9(0.6-1.2)
Rusted	2.3(1.6-2.9)	0.8(0.5-1.3)	1.3(0.8-1.8)	0.8(0.5-1.3)

Therefore, we can apply the shell model to describe the mitral valve. We can produce the 3D prosthetic valve of the human mitral valve by 3D printing technology as long as we choose suitable biological materials. Furthermore, we will simulate the physiological function of the human mitral valve by a suitable dynamic physical equation.

## Results and discussion

In this paper, we propose a geometric model for the the human mitral valve based on its statistical data. First, we studied the human mitral valve as an elastic shell according to its structure and size. Based on the morphological characteristics of the mitral valve during its entire opening, we divided it into the anterior and the posterior parts, each of which were treated as a portion of the semi-elliptic cylindrical shell and were provided with parametric equations, respectively. Secondly, based on the available and specific medical image data of the human mitral valve, we chose the appropriate fitting parameters and visualized the fitting results. Finally, we showed the accuracy of the fitting function by comparing the fitting results of our parametric equations with the statistical data.

Based on the fitting shape, the authors plan to further study the function simulation of the human mitral valve by a suitable dynamic physical equation.

## Supporting information

S1 Fig The mitral annulus. It was plotted by Microsoft office visio on the basis of the size of the mitral annulus in reference [14], and we offered the source file “S1_Fig.vsd” for Fig 1.
(VSD)

S2 Fig Diagram of the heart valve. It was plotted by Microsoft office visio on the basis of the data in Table 1, and we offered the source file “S2_Fig.vsd” for Fig 2.
(VSD)

S3 Fig The anterior leaflet. It was plotted by Matlab on the basis of the size of the anterior leaflet in Table 1, and we offered the source file “S3_Fig.m” for Fig 3.
(M) Click here for additional data file.

S4 Fig The boundary curve of the anterior leaflet. It was plotted by Microsoft office visio on the basis of Fig 3, and we offered the source file “S4_Fig.vsd” for Fig 4.
(VSD)

S5 Fig The posterior leaflet. It was plotted by Matlab on the basis of the size of the posterior leaflet in Table 1, and we offered the source file “S5_Fig.m” for Fig 5.
(M) Click here for additional data file.

S6 Fig The boundary curve of the posterior leaflet. It was plotted by Microsoft office visio on the basis of Fig 5, and we offered the source file “S6_Fig.vsd” for Fig 6.
(VSD)

S7 Fig Visualization results. They were coded by FreeFem++ and visualized by Paraview, and we offered the FreeFem++ code file “S7_Fig.edp” for Fig 7.
(EDP)
